# Myelitis Caused by Infection of *Angiostrongylus cantonensis*

**DOI:** 10.4269/ajtmh.2010.10-0418

**Published:** 2010-12-06

**Authors:** Zongli Diao, Chenghong Yin, Erhu Jin

**Affiliations:** Beijing Tropical Medicine Research Institute, Beijing Friendship Hospital, Capital Medical University, Beijing, China; Department of Radiology, Beijing Friendship Hospital, Capital Medical University, Beijing, China

**Figure 1.**
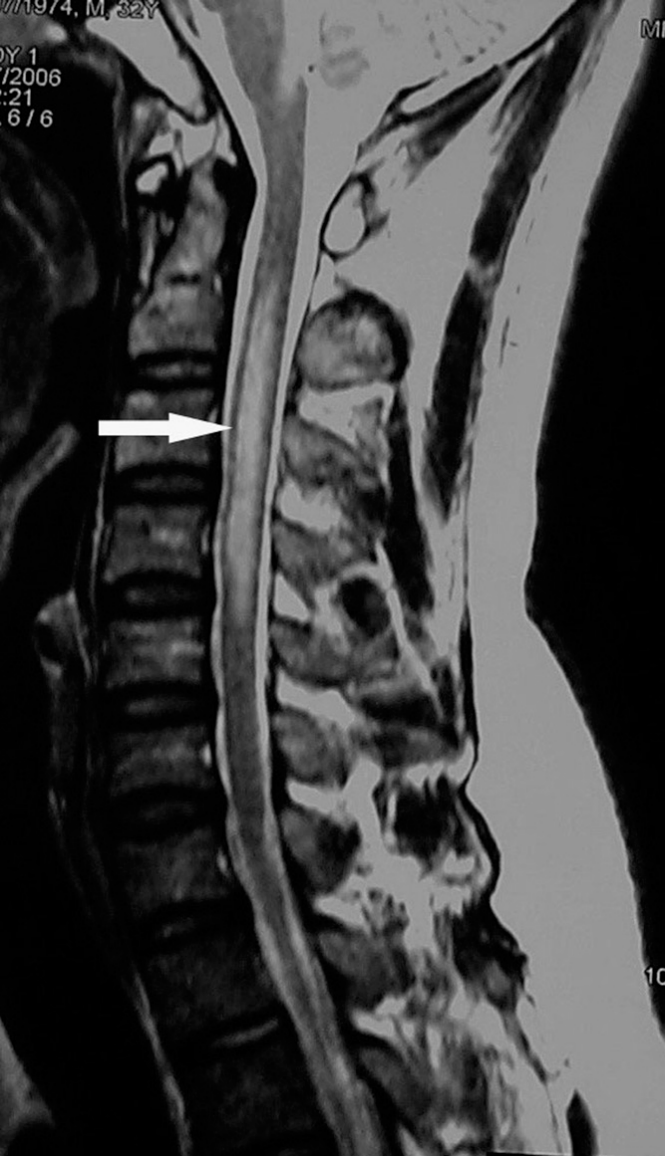
A lesion in the cervical spinal cord presented as hyperintense on a sagittal T2WI.

**Figure 2.**
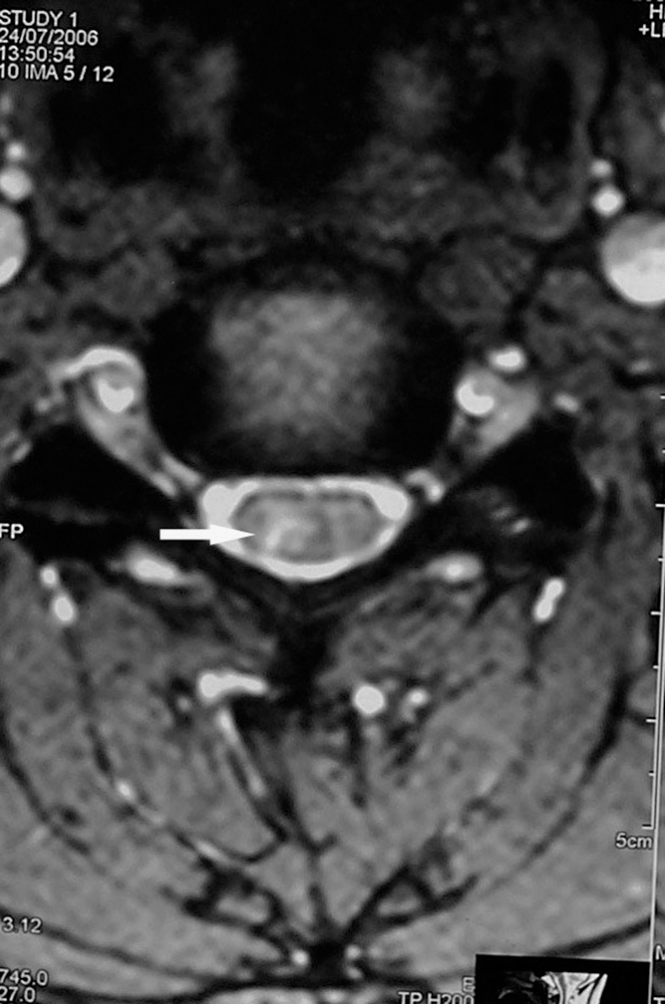
A lesion in the cervical spinal cord presented as hyperintense on a transverse T2WI.

A 32-year-old man presented to our hospital on July 5, 2006, after the onset of headache, paresthesias of the left upper limb for 10 days, and weakness for 7 days before admission. He had eaten an inadequately cooked *Pomacea canaliculata* 20 days previously. Laboratory testing indicated a normal white blood cell count of 6,700/mm^3^ with mild eosinophilia of 7.8% (523/mm^3^). A lumbar puncture test showed an opening pressure of 220 mm H_2_O and 160 cells with 23% eosinophils, and cerebrospinal fluid (CSF) cultures were negative. We detected the circulating antigens (CAg) of *Angiostrongylus cantonensis* by double antibody sandwich enzyme-linked immunosorbent assay (ELISA), and they tested positive. This method had a high sensitivity (86.4%), and no cross-reactions with sera from patients with many other parasites were observed.^1^ Therefore, the result was helpful for diagnosis. Spinal magnetic resonance imaging (MRI) showed a lesion with high signal intensity in the cervical spinal cord on both sagittal and transverse T2-weighted imaging (T2WI) (Figures 1 and 2) at 9 days after admission.

**Figure 3.**
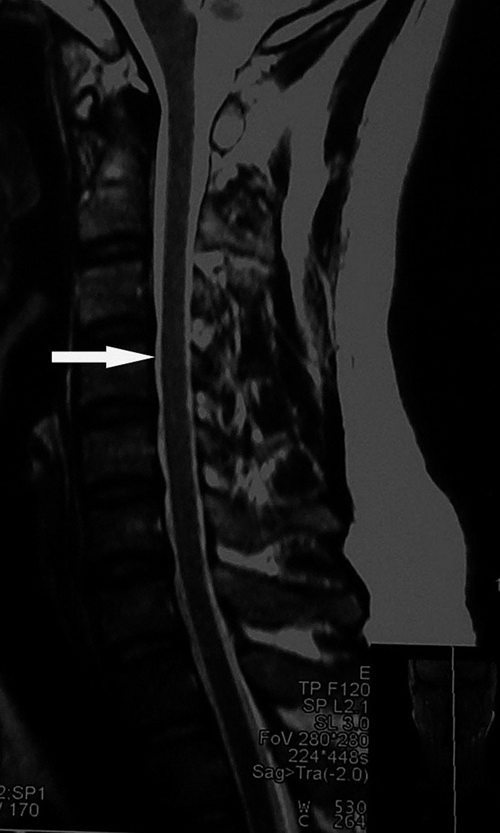
The abnormally high signal on a sagittal T2WI completely disappeared.

**Figure 4.**
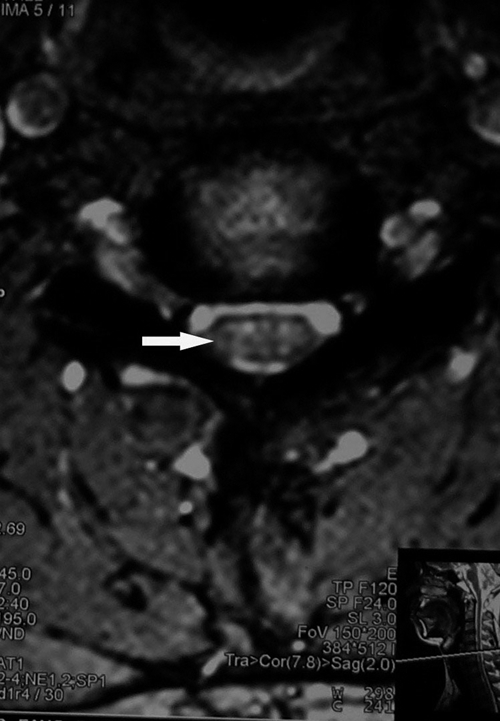
The abnormally high signal on a sagittal T2WI completely disappeared.

On the basis of history, clinical presentation, and examinations, a diagnosis of angiostrongyliasis was made,^2^ and the patient was treated with a combination of albendazole and dexamethasone. Symptoms of headache and paresthesia resolved within 14 days, and spinal-cord lesions completely resolved by a 1-month follow-up (Figures 3 and 4).
